# Fetomaternal Outcomes and Associated Factors among Mothers with Hypertensive Disorders of Pregnancy in Suhul Hospital, Northwest Tigray, Ethiopia

**DOI:** 10.1155/2022/6917009

**Published:** 2022-11-09

**Authors:** Fisseha Hailemariam Syoum, Girmatsion Fisseha Abreha, Dessalegn Massa Teklemichael, Mebrahtu Kalayu Chekole

**Affiliations:** ^1^Tigray Regional Heath Bureau, Mekelle, Ethiopia; ^2^School of Public Health, College of Health Science, Mekelle University, Mekelle, Ethiopia

## Abstract

**Background:**

Hypertensive disorder of pregnancy is the leading cause of maternal and perinatal morbidity and mortality worldwide and the second cause of maternal mortality in Ethiopia. The current study is aimed at assessing fetal-maternal outcomes and associated factors among mothers with hypertensive disorders of pregnancy complication at Suhul General Hospital, Northwest Tigray, Ethiopia, 2019. *Methods:*A hospital-based cross-sectional study was conducted from Oct. 1^st^, 2019, to Nov. 30, 2019, at Suhul General Hospital women's chart assisted from July 1^st^, 2014, to June 31^st^, 2019. Charts were reviewed consecutively during five years, and data were collected using data abstraction format after ethical clearance was assured from the Institutional Review Board of Mekelle University College of Health Sciences. Data were entered into Epi-data 3.5.3 and exported to SPSS 22 for analysis. Bivariable and multivariable analyses were done to ascertain fetomaternal outcome predictors. Independent variables with *p* value < 0.2 for both perinatal and maternal on the bivariable analysis were entered in multivariable logistic regression analysis and the level of significance set at *p* value < 0.05.

**Results:**

Out of 497 women, 328 (66%) of them were from rural districts, the mean age of the women was 25.94 ± 6.46, and 252 (50.7%) were para-one. The study revealed that 252 (50.3%) newborns of hypertensive mothers ended up with at least low Apgar score 204 (23.1%), low birth weight 183 (20.7%), preterm gestation 183 (20.7%), intensive care unit admissions 90 (10.2%), and 95% CI (46.1% -54.9%), and 267 (53.7%) study mothers also developed maternal complication at 95% (49.3-58.1). Being a teenager (AOR = 1.815: 95%CI = 1.057 − 3.117), antepartum-onset hypertensive disorders of pregnancy (AOR = 7.928: 95%CI = 2.967 − 21.183), intrapartum-onset hypertensive disorders of pregnancy (AOR = 4.693: 95%CI = 1.633 − 13.488), and low hemoglobin level (AOR = 1.704: 95%CI = 1.169 − 2.484) were maternal complication predictors; rural residence (AOR = 1.567: 95%CI = 1.100 − 2.429), antepartum-onset hypertensive disorders of pregnancy (AOR = 3.594: 95%, CI = 1.334 − 9.685), and intrapartum-onset hypertensive disorders of pregnancy (AOR = 3.856: 95%CI = 1.309 − 11.357) were predictors of perinatal complications.

**Conclusions:**

Hypertensive disorder during pregnancy leads to poor fetomaternal outcomes. Teenage age and hemoglobin levels were predictors of maternal complication. A rural resident was the predictor of poor perinatal outcome. The onset of hypertensive disorders of pregnancy was both maternal and perinatal complication predictors. Quality antenatal care services and good maternal and childcare accompanied by skilled healthcare providers are essential for early detection and management of hypertensive disorder of pregnancy.

## 1. Introduction

Hypertension during pregnancy is defined as a blood pressure of greater than or equal to 140 mmHg (systolic) or 90 mmHg (diastolic) on at least two measurements of four hours [1]. Hypertensive disorder of pregnancy (HDP) complicates 10% to 15% of all pregnancies and leads to maternal and perinatal mortality and morbidity worldwide [2–4].

HDP is unknown with its etiology; it is a multisystem disease with a heterogeneous nature and variable progression [5]. Secular increases in HDP have occurred as a result of changes in maternal characteristics, whereas declines in eclampsia have followed widespread antenatal care (ANC) and use of prophylactic treatments such as magnesium sulphate [6]. HDP is associated with an increased risk of adverse fetal, neonatal, and maternal outcomes including renal or hepatic failure, hemorrhage, and stroke [3, 7]. Predicting the onset of these complications could aid in for better management and good outcome of both for the mother and fetus and reduces morbidity and mortality from the HDP [5, 8]. Institutional care with close maternal monitoring, prevention and control of seizures through the use of anticonvulsants, treatment of severe hypertension, and timely termination of pregnancy reduces maternal mortality and serious morbidity [9]. Favorable maternal and fetal outcomes are dependent on the health-seeking behavior and infrastructure including the three delays which are avoidable factors but contribute to worsening of maternal outcomes with HDP [10].

Millions of women develop preeclampsia each year around the world, which is a cause of maternal and perinatal mortality and morbidity [10, 11]. HDP complicates 5.2%-8.2% of pregnancies globally [12]. The World Health Organization (WHO) estimates the incidence of preeclampsia to be seven times higher in low-income nations (2.8% of live births) than in high-income nations (0.4%) [13].

In a systemic review of the WHO (2014), HDP is associated with about 16% of maternal mortality and is the leading cause of maternal death (after hemorrhage) in Sub-Saharan Africa [14, 15]. Similarly, the national maternal death surveillance and response (MDSR) of Ethiopia revealed that HDP (19%) is the direct cause of maternal deaths after hemorrhage and with a perinatal mortality of 111.1 per 1000 live births [16–18].

In improved infrastructure, hypertensive maternal outcomes have resulted from good access and high-quality care for all pregnant women and early detection and timely action of a syndrome [19]. Access to good quality basic emergency obstetric care is a key strategy to improve maternal outcome with HDP, which would prevent 50% to 70% of maternal deaths, and neonatal mortality (10% to 15%) and substantially reduce a sequel of obstetric complications [20, 21].

Ethiopia follows the newest WHO antenatal care policy, to promote safe pregnancies, to prevent, and to manage problems [22]. Moreover, MDSR was launched to mitigate the challenges owing to delay in receiving quality maternal health services [23].

Consciousness of healthcare providers plays a vital role in the control of preeclampsia in low-income countries [24]. In Ethiopia, even though blood pressure measurement and urine tests for protein urea are among the components of routine ANC service, the 2016 Ethiopian Demographic and Health Survey (EDHS) report indicates 62% and 31% of pregnant women had at least one and fourth ANC visits, respectively, and 75% of pregnant women had their blood pressure measured; 66% had a urine test and nutritional counseling and shortage of investigation modalities, medications, and actual services provided to treat the HDP [22, 25]. National evidence also shows that most health centers were weak in providing life-saving Basic Emergency Obstetric and Newborn Care (BEmONC) interventions; service is limited to city centre [26]. Therefore, the current study was aimed at filling the gap by assessing the magnitude of the adverse outcomes of HDP and determined the predicting factors of adverse outcomes of HDP at Suhul Hospital, Northwest Tigray Region.

Prediction of adverse maternal outcomes from the HDPs helps for better management, increased surveillance, treatment of symptoms, transfer to the higher care facility, improving the design of clinical trials, and filling the gap in the adapted intervention in Ethiopia. The finding of this study has both clinical and public intervention importance in addressing the problems associated with adverse outcomes of HDP.

## 2. Methodology

The study was conducted at Suhul Hospital, which is found in Shire town in Northwest of Tigray, Ethiopia. Maternity unit is the one which gives inpatient and outpatient services with 50 beds as well as three coaches. There are two emergency trained obstetricians, one gynecologist, and twelve midwives working in the obstetric and genecology ward. The data was collected from October 1^st^, 2019, to November 30, 2019, and an institutional-based 5-year retrospective cross-sectional study was applied to answer the research objective. All (526) pregnant mothers who had HDP and gave birth after 20 weeks of gestation in hospital over a five-year period were included in the study, and maternal charts with incomplete data and twin gestation were excluded from the study.

After calculating for both objectives, the sample size for the 1^st^ objective was 384 by considering 95% CI, 5% marginal error, and 50% of prevalence because the prevalence of maternal outcome of mothers who develop HPD during pregnancy in Ethiopia was unknown. Then, we used 10% contingency and the final sample size was 423 charts. The sample size for the 2^nd^ objective was also calculated by using double population proportion; mothers with unfavorable neonatal outcomes with estimated proportion of 46.5% in Amhara region [27] and mothers with HDP were taken to get the maximum sample size 382. But the total number of women with HDP was 526 in which we can handle it financially and decided to use the whole registration cards. So, all the medical registration numbers (MRN) of mothers with HDP classification were extracted within five years (July 1^st^, 2014, to June 31^st^, 2019). A five-year (July 1^st^, 2014, to June 31^st^, 2019) delivery registration book and maternity in patient diagnosis registration were used to collect the medical registration number of mothers (MRN) with HDP in consecutive five years, and maternal registration number was used to retrieve the individual maternal charts. So that the final sample size was 497 from 526 of mothers with HDP, the rest of the charts are excluded: 14 incomplete, 6 of them were twins, and 9 of them did not appear in the catalog of the data record office.

A structured data abstraction format was prepared by reviewing different literatures and was used to abstract data from the included mothers' charts. Data was collected by record reviewing of maternal charts; registration books and delivery log book were used for newborn information. Background information was as follows: maternal age, address, source of referral, prereferral medication, and parity. Obstetric characteristics were as follows: history of stillbirth, gestational age, parity, onset of HDP, antenatal care visit, birth weight, and diagnosis at admission. Clinical and laboratory data include systolic blood pressure, diastolic blood pressure, platelet count, and liver and renal function results. Pregnancy complications and outcomes, onset of labor, treatment given, mode of delivery, birth weight, maternal outcome, and fetal outcome were collected by one BSc midwifery, one BSc nurse, and one BSc health officer under the supervision of principal investigators. Quality was ensured by giving two-day training to data collectors and supervisors. Pretesting was done, and checklist was checked by a principal investigator after data collection; data were cleaned and coded before data entry.

Data were entered to Epi-data version 3.5.3 then exported to SPSS version 22 for statistical analysis. Summary statistics were computed for description, and variables that were found significant in the bivariable logistic regression with *p* < 0.2 cut-off point for both perinatal and maternal adverse outcomes were entered to the final multivariable model to adjust for confounders. Newborn and mother with at least one poor outcome were transformed as dependent to perinatal and maternal model, respectively. Finally, the odds ratio at 5% confidence interval was reported and statistical significance of the association was declared at a *p* < 0.05 at 95% CI to identify associated factors on maternal and perinatal outcomes.

The Ethical Review Committee of Mekelle University, College of Health Sciences, approved the study protocol, and support letter was obtained from Tigray Regional Health Bureau. Any personal identifier was not encoded; identifiers of the women were replaced with identification numbers. All the data taken from the files were considered highly confidential, and patient names were avoided during data collection.

## 3. Result

### 3.1. Sociodemographic and Obstetric Characteristics of Mothers

A total of 497 hypertensive mothers were included in the study at which 328 (66%) of the mothers were from rural district. Above half of participants, 252 (50.7%), were primigravida and 476 (95.7%) had at least one ANC contact. The mean age of mothers was 25.94 (SD ± 6.46) years in which 69.8% of them were within the age group 20-34 years. The median gestational age of mothers was 37 weeks ranging from 35 to 39 weeks, and 91 (18.3%) of the mothers had poor obstetric history. Only 378 (76%) had a source of referral, and 62 (82%) of the adolescent were from rural districts. Fourteen (2.8%), 6 (1.2%), 4 (0.8%), and 2 of the hypertensive mothers were having anemia, malaria, urinary tract infection, and STIs, respectively, during admission (Table 1).

### 3.2. Condition of the Mothers at Admission and Labor

**Figure 1:**
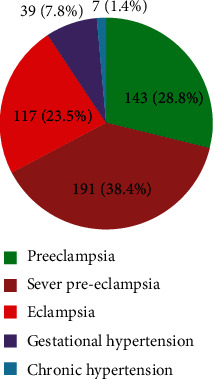
Maternal diagnosis during admission among mothers with HDP from July 01, 2014, to June 31, 2019, at Suhul Hospital, Northwest Tigray, Ethiopia, 2019.

Based on the result of the study, mothers developed severe forms of HDP at admission; the figure of HDP of mothers is as follows: 117 (23.5%) eclamptic, 191 (38.4%) severe preeclamptic, 143 (28.8%) preeclampsia, 39 (7.8%) gestational hypertension, and 7 (1.4%) superimposed; 308 (61.97%) were referred from the rural areas (Figure 1).

Majority 380 (76.4%) of mothers had antepartum onset of hypertensive disorder, and 285 (57.3%) hypertensive mothers were induced to start labor or terminate pregnancy. But around 63 (22%) of the induced pregnant women failed the induction. Out of the total deliveries, 297 (59.8%) took place by spontaneous vaginal delivery and 131 (26.4%) cesarean section mainly for failed induction, better management, and malpresentation. From the total induced mothers, 162 (57%) give birth though the vagina without any instrumental support. The median highest systolic BP was 154 mmHg which ranges from 145 to 166mmHg. Among the hypertensive mothers, 183 (37%) of them give low birth infant and the mean weight of the newborns was 2565.69 (SD ± 747.4 grams) (Table 2). The proportion of stillbirth was 37 (68.5%), 9 (16.7.0%), and 8 (14.8%) with instrumental, vaginal, and cesarean section mode of delivery, respectively. The respective diagnoses of teenagers were 38 (50%) eclampsia, 23 (30.3%) severe preeclampsia, 13 (17.1%) preeclampsia, and 2 (2.6%) superimposed at admission. The proportion of perinatal mortality with respect to maternal gestational age was 61 (42.6%), 37 (25.9%), and 45 (31.4%) among very preterm, preterm, and term mothers, respectively. Around 231 (46.4%) mothers were given magnesium sulphate (MgSO_4_) loading dose before referrals at their respective health facility, and 427 (86%) mothers were also given anticonvulsant during or postdelivery after admission.

### 3.3. Fetal Outcomes of Hypertensive Disorders of Pregnancy

**Figure 2:**
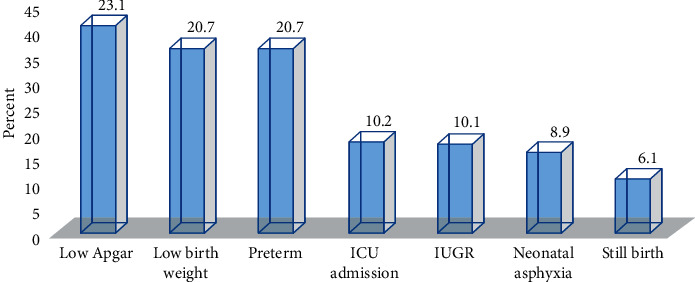
Magnitude of neonatal complications among hypertensive mothers at Suhul Hospital, Northwest Tigray, Ethiopia, 2019.

The study finding showed that half of 250 (50.3%) newborns of the hypertensive mothers ended up having 204/882 (23.1%) low Apgar score, 183/882 (20.7%) low birth weight, 183/882 (20.7%) preterm, 90/882 (10.2%) ICUA, 89/882 (10.1%) IUGR, 79/882 (8.9%) neonatal asphyxia, and 54/882 (6.1%) stillbirth with 95% CI (46.1%─54.9%) out of the total complications (Figure 2).

The proportion perinatal complications in the type of HDP was 505/882 (57.3%), 490/882 (55.5%), 352/882 (39.9%), and 226/882 (25.6%) among eclampsia, severe preeclampsia, preeclampsia, and gestational hypertension, respectively. The relative frequency of perinatal complication with gestational age was 205 (82%) preterm and 45 (29.6%) term gestation. The magnitude of complication relative to maternal parity was 445/882 (50.4%) para one, 498/882 (56.5%) para two, and 370/882 (42%) multipara. The distribution of low birth weight relative to gestational age was very preterm, preterm, and term gestation (699 (79.2%), 517 (58.6%), and 168 (19%)), respectively. From the total perinatal death, preterm and low birth weight (77% and 70%) were the leading, respectively (Figure 2).

### 3.4. Maternal Adverse Outcomes of Hypertensive Disorders of Pregnancy

Among the hypertensive mothers, 267 (53.7%) of them were having one of HDP: 131 (26.3%) cesarean section due to HDP, 86 (17.3%) length of stay > 7 days, 12 (2.4%) antepartum hemorrhage, 11 (2.2%) postpartum hemorrhage, 10 (2%) maternal death, and 8 (1.6%) generalized body swelling at 95% (49.3−58.1). Cesarean section was taken as a solution to the most frequent adverse outcome observed which accounts to 47% the complications. The percentage of maternal complication in the respective type of diagnosis was 68 (58.1%) eclampsia, 105 (55%) severe preeclampsia, 20 (51.3%) gestational hypertension, and 71 (49.7%) preeclampsia mothers who develop complication. Likewise, relative to onset of HDP were 223 (58.7%) antepartum, 39 45.9% intrapartum, and 5 (15.6%) postpartum-onset mothers who developed poor outcomes. Sixty-one (32%) severe preeclampsia and 16 (14%) eclampsia mothers delivered by cesarean section.

### 3.5. Factors Associated with Perinatal and Maternal Adverse Outcome

During the bivariable logistic regression analysis, descriptive statistics and odds ratio with 95% confidence interval were calculated to see related predictor variables with perinatal and maternal adverse outcomes and associated predictor variables. *p* value < 0.2 cut-off point was entered to multivariable logistic in both fetal and maternal, and the model fitness of Hosmer-Lemeshow test (HL test) result was 0.52 for perinatal and 0.47 maternal models, respectively. After multivariable logistic regression analysis, newborns from rural resident mothers were two times more likely to have fetal complication than the urban (AOR = 1.567: 95%CI = 1.1001 − 2.429). Newborns from mothers with antepartum onset of hypertension were also four times more likely to have perinatal complication (AOR = 3.594: 95%CI = 1.334 − 9.685) than those with postpartum onset. Similarly, newborn from mothers with intrapartum onset of HDP was four times more likely to have poor fetal outcome than mothers with postpartum onset (AOR = 3.856: 95%CI = 1.309 − 11.357).

After controlling for confounding effect of different variables, teenager mothers were two times more likely to have to adverse maternal outcome (AOR = 1.815: 95%CI = 1.057 − 3.117). Mothers with antepartum onset of hypertension were eight times more likely to have maternal complication (AOR = 7.928: 95%CI = 2.967 − 21.183) and mothers with intrapartum onset were five times more likely to have maternal complication (AOR = 4.693 : 95%CI = 1.633 − 13.488) than mothers with postpartum onset. Low hemoglobin level was associated with poor maternal adverse outcome (AOR = 1.704: 95%CI = 1.169 − 2.484) than not anemic mothers (Table 3).

## 4. Discussions

This study was aimed at assessing the magnitude of fetomaternal outcomes and factors associated with unfavorable fetal and maternal outcomes among women with hypertensive disorders of pregnancy. This hospital-based study showed that the magnitude of maternal adverse outcome was 53.7% and the magnitude of fetal adverse outcome was 50.3% among the hypertensive mothers and 51% participants were primigravida. Teenage hemoglobin level was an independent predictor of maternal complication. Rural residence was the predictor factor of unfavorable perinatal outcomes of the hypertensive mothers. Antepartum and intrapartum onset of HDP was found to be a predictor of both maternal and perinatal adverse outcomes.

The magnitude of maternal complications in the study was higher than that in a study conducted in India and Saudi Arabia [28, 29]. This may be due to difference in health-seeking behavior, access to roads, or transport as well as getting optimum management, early identification of high risk women, and postpartum follow-up. This study was the first study conducted in an institution based in Ethiopia, and it is considered as the strength of the study specific to maternal outcome.

The magnitude of the perinatal complications in the current study is in line with the study conducted in Amara region (46.5%). But a study lower than a study done in Addis Ababa [27, 30] was found higher than the study done in India [29]. The implication that the current study is in line with the study done in Amara is that it may be due to the similarity of age distribution of participants where in both studies, 70% of the mothers fall from 20 to 34 years or may be the closeness in operationalizing variables to measure the magnitude and being both recent evidence while the difference could be the study setting where the study in Addis Ababa was at referral hospitals with better diagnosis and classification or may be due to improvement in healthcare system access (introducing the CEmONC) and maternal service utilization recently and explanation of health facilities, while the difference with India may be due to maternal care provision and quality of critical care units.

The odds of having maternal complication were twofold among the teenagers than adult mothers. It is similar with a study done in Nigeria [31] and the USA [32]. This is also due to the fact that teenage pregnancy trebles the risk for developing eclampsia [33].

Hypertensive mothers with antepartum onset were eight times more to develop maternal complication than those with postpartum onset. This study also agrees with another study at Gandhi Memorial Hospital Addis Ababa [34]. This may be due to the three delays that may complicate the hypertensive disorder. Also, mothers with intrapartum onset of HDP were five times more likely to have maternal complication than those with postpartum onset. This is supported with the fact that termination of pregnancy leads to rapid progression of preeclampsia diseases often without residual effects [9]. It may be due to failure to follow clinical protocols of care, arrivals of most women as an emergency event, failure to achieve hemodynamic stabilization, and mode of delivery.

On the other hand, anemic mothers were two times more likely to have maternal complication than nonanemic mothers and it is the same with the study done in Sudan [35]. This may be because severe anemia caused by malaria is a major factor in those outcomes. A literature also indicated that malaria increases the risk of hypertensive disorder during pregnancy [36]. It might be difficult to determine if the maternal adverse outcomes among the anemic were caused by hypertensive disorders of pregnancy or effect of underlying anemia and the existing fact that severe anemia causes preeclampsia and eclampsia [37]. None of the mothers were diagnosed as having HELLP syndrome in this study; as result of this, it may need further study.

For fetal predictors, newborns with mothers referred from the rural resident were two times more to have fetal complication than those with mothers from urban. It is in line with a study at Addis Ababa [38] and in a group of participants from British Columbia and Canada [39]. This may be due to the late initiation of management, delayed arrival mothers to hospital, poorly equipped ambulance system, and distance with poor infrastructure mothers from the rural districts than the urban mothers.

Newborns from hypertensive mothers with antepartum onset of HDP were four times more likely to develop fetal complication than those with postpartum onset. This is in line with study done in south Ethiopia [40] and mothers with intrapartum onset. This could be a delay in diagnosis and delay in providing treatment in the early stage of the disease and may be the three delays, and it may be due to poor monitoring of mother, delivery mode, weak neonatal service, and poor control of blood pressure.

## 5. Limitation of the Study

The study is limited by its retrospective nature and its dependence on patients' records in all women. Furthermore, this hospital-based approach includes only women attending the hospital, as many women die in rural area without visiting a health facility, as well as the perinatal death was considered only for these at an institution. Women may not be evaluated (investigated) for many of the adverse outcomes.

## 6. Conclusion

This hospital-based study revealed that hypertensive disorder during pregnancy leads to poor fetomaternal outcomes. Teenage age and hemoglobin levels were predictors of maternal complication. A rural resident was the predictor of poor perinatal outcome. The onset of hypertensive disorders of pregnancy was both maternal and perinatal complication predictors. Quality antenatal care services and good maternal and childcare accompanied by skilled healthcare providers are essential for early detection and management of hypertensive disorder of pregnancy.

## Figures and Tables

**Table 1:** Sociodemographic and obstetric characteristics of mothers with hypertensive disorders of pregnancy at Suhul Hospital, Northwest Tigray, Ethiopia, 2019.

Variable	Category	Frequency	Percent
Residence	Urban	169	34.0
	Rural	328	66
	Total	497	100.0

Maternal age	≤19 years	76	15.3
	20˗34	347	69.8
	≥35	74	14.9

	Mean (±SD)	25.94 (±6.46)	
Obstetric history	Stillbirth	13	2.6
	Abortion	69	13.8
	Preterm	2	.04
	Preeclampsia	7	1.4
	Healthy	406	81.6

Medical illness	Yes	26	5.23
	No	471	94.8

Source of referral	Yes	378	76.1
	No	119	23.9

Parity	Para one	252	50.7
	Para two	69	13.9
	Para ≥ 3	176	35.4

Gestational age (weeks)	20─33 weeks	72	14.5
	34─36	111	22.3
	Term	314	63.2

	Median of gestational age	37 IQR (2) weeks	
ANC attendance	Yes	476	95.7
	No	21	4.3

Nausea and vomiting	Yes	99	19.9
	No	398	80.1

**Table 2:** Characteristics of mothers with HDP during admission and labor from July 01, 2014, to June 31, 2019, at Suhul Hospital, Northwest Tigray, Ethiopia, 2019.

Characteristics	Frequency	Percentage
On set of HDP		
Antepartum	380	76.4
Intrapartum	85	17.1
Postpartum	32	6.4
Onset of labor		
Spontaneous	143	28.8
Induced	285	57.3
Direct C/S	69	13.9
Mode of delivery		
Vaginal	297	59.8
Instrumental	69	13.9
Cesarean section	131	26.3
Highest systolic BP		
<140 mmHg	43	8.7
140–159 mmHg	245	49.3
≥160 mmHg	209	42.1
Highest diastolic BP		
<90 mmHg	47	9.5%
90–109 mmHg	308	61.9%
≥110 mmHg	142	28.6
Hemoglobin level (gm/dL)		
<10	63	12.7
10−11.9	139	28
≥12	295	59.3
Birth weight gram		
≥2500	314	63.2
<2500	183	36.8
Apgar score at 5^TH^ minute		
≥7	385	77.5
<7	112	22.5

**Table 3:** Binary and multivariable logistic regression results of factors associated with fetomaternal outcomes among women with hypertensive disorders of pregnancy at Suhul General Hospital, Northwest Tigray, Ethiopia, from July 01, 2014, to June 31, 2019.

Variables	Category	Unfavorable	Favorable	COR (95% CI)	AOR (95% CI)
		Yes *N* (%)	No *N* (%)		
Predictors of fetal outcomes among women with hypertensive disorders of pregnancy					
Residence	Rural	168 (51.9)	156 (48.1)	1.407 (0.971─2.040)	1.567 (1.1001−2.429)^∗^
	Urban	75 (43.4)	98 (56.6)	1	
Parity of mother	Para one	130 (51.6)	122 (48.4)	1.469 (0.996−2.165)	1.516 (0.960─2.394)
	Para two	39 (56.5)	30 (43.5)	1.792 (1.021─3.114)^∗^	1.849 (0.937─3.646)
	Para ≥ 3	74 (42)	102 (58)	1	
On set of HDP	Antepartum	196 (51.6)	184 (48.4)	3.804 (1.607─9.008)^∗∗^	3.594 (1.334─9.685)^∗^
	Intrapartum	40 (47.1)	45 (52.9)	3.175 (1.240─8.128)^∗^	3.856 (1.309─11.357)^∗^
	Postpartum	7 (21.9)	25 (78.1)	1	
Highest systolic BP	<140 mmHg	17 (39.5)	26 (60.5)	1	
	140–159	114 (46.5)	131 (53.5)	1.331 (0.687─2.577)	0.983 (0.469─2.059)
	160+	112 (53.6)	97 (46.4)	1.766 (0.905─3.448)	0.872 (0.378─1.931)
Highest diastolic BP	<90 mmHg	18 (38.3)	29 (61.7)	1	
	90–109	137 (44.5)	171 (55.5)	1.291 (0.688─2.423)	1.330 (0.634─2.792)
	110+	88 (62)	54 (38)	2.626 (1.332─5.176)^∗∗^	1.990 (0.895─4.428)
Mode of delivery	Vaginal	186 (50.8)	180 (49.2)	1.342 (0.898─2.005)	1.156 (0.718─1.861)
	C/S	57 (43.5)	74 (56.5)	1	
Predictors of maternal outcome among women with hypertensive disorders of pregnancy					
Maternal age	≤19 years	48 (63.2)	28 (36.8)	1.704 (1.022─2.842)^∗^	1.815 (1.057─3.117)^∗^
	20─34	174 (50.1)	173 (49.9)	1	
	≥35 years	45 (60.8)	29 (39.2)	1.543 (0.925─2.574)	1.528 (0.901─2.593)
Nausea and vomiting	Yes	60 (60.6)	39 (39.4)	1.420 (0.906─2.223)	
	No	207 (52)	191 (48)	1	1.430 (0.884─2.311)
Onset of HDP	Antepartum	223 (58.7)	157 (41.3)	7.670 (2.891−20.352)^∗∗^	7.928 (2.967─21.183)^∗∗^
	Intrapartum	39 (45.9)	46 (54.1)	4.578 (1.610─13.021)^∗∗^	4.693 (1.633─13.488)^∗∗^
	Postpartum	5 (15.6)	27 (84.4)	1	
Hemoglobin level (gm/dL)	<12	124 (61.4)	78 (38.6)	1.690 (1.174─2.431)^∗∗^	1.704 (1.169−2.484)^∗∗^
	≥12	143 (48.5)	152 (51.5)	1	

## Data Availability

Our data will not be shared in order to protect the participants' anonymity.

## Abbreviations

ANC: Antenatal care
BEmONC: Basic Emergency Obstetric and Newborn Care
EDHS: Ethiopian Demographic Health Survey
HDP: Hypertensive disorders of pregnancy
HELLP: Hemolysis, elevated liver enzymes, and a low platelet count
IUGR: Intra uterine growth retardation
MRN: Medical registration number
SPSS: Statistical Package for Social Sciences
WHO: World Health Organization.
